# Exponentially fitted multisymplectic scheme for conservative Maxwell equations with oscillary solutions

**DOI:** 10.1371/journal.pone.0256108

**Published:** 2021-08-27

**Authors:** Xiuling Yin, Yanqin Liu, Jingjing Zhang, Yanfeng Shen, Limei Yan

**Affiliations:** 1 School of Mathematics and Big Data, Dezhou University, Dezhou, China; 2 School of Sciences, East China Jiaotong University, Nanchang, China; Max-Planck-Institut fur Mathematik in den Naturwissenschaften, GERMANY

## Abstract

Aiming at conservative Maxwell equations with periodic oscillatory solutions, we adopt exponentially fitted trapezoidal scheme to approximate the temporal and spatial derivatives. The scheme is a multisymplectic scheme. Under periodic boundary condition, the scheme satisfies two discrete energy conservation laws. The scheme also preserves two discrete divergences. To reduce computation cost, we split the original Maxwell equations into three local one-dimension (LOD) Maxwell equations. Then exponentially fitted trapezoidal scheme, applied to the resulted LOD equations, generates LOD multisymplectic scheme. We prove the unconditional stability and convergence of the LOD multisymplectic scheme. Convergence of numerical dispersion relation is also analyzed. At last, we present two numerical examples with periodic oscillatory solutions to confirm the theoretical analysis. Numerical results indicate that the LOD multisymplectic scheme is efficient, stable and conservative in solving conservative Maxwell equations with oscillatory solutions. In addition, to one-dimension Maxwell equations, we apply least square method and LOD multisymplectic scheme to fit the electric permittivity by using exact solution disturbed with small random errors as measured data. Numerical results of parameter inversion fit well with measured data, which shows that least square method combined with LOD multisymplectic scheme is efficient to estimate the model parameter under small random disturbance.

## Introduction

Maxwell equations are basic and important mathematical physical models in electromagnetism. They reflect the general law of electromagnetic field excited by charge current and the internal motion of electromagnetic field. The study of the equations is helpful to deepen and enrich the understanding of the materiality of electromagnetic field. Efficient numerical analysis to Maxwell equation is an important study hotspot. Numerical investigations of Maxwell equations include symplectic exponential integrator [1], compact scheme [2], Runge-Kutta method [3], stochastic Euler scheme [4], multisymplectic schemes [5–8], energy-conserving method [9], wavelet collocation method [10, 11], Hamiltonian time integrators [12], finite element method [13, 14], spectral method [15] and other numerical methods.

In the past few decades, multisymplectic schemes, as a kind of structure-preserving algorithms, are popular and gain wide applications in scientific computing due to long-time stability in numerical simulation [5–8, 11, 16–19]. A review on stochastic multisymplectic schemes for Maxwell equations was given by Zhang et al. in [5]. Cui et al. [17] presented stochastic multisymplectic method to solve random Schrödinger equation. Hong et al. [6, 7] proposed multisymplectic methods and investigated discrete conservative quantity to stochastic Maxwell equations driven by additive noise. Multisymplectic wavelet collocation method for Maxwell’s equations were considered in [10, 11]. Zhu et al. [18] also applied multisymplectic wavelet collocation methods to solve Schrödinger equations. High-order compact multisymplectic method was presented to simulate Schrödinger equations in [19]. Kong et al. [8] also studied splitting multisymplectic integrators for Maxwell’s equations. Since much attention has been gained in multisymplectic schemes, we are committed to the investigations on multisymplectic schemes to simulate Maxwell equations with oscillatory solutions.

Numerical method is a promising tool to solve oscillatory differential equations. Some recent works can be found in [20–29]. For example, Wu et al. summarized structure-preserving algorithms for oscillatory differential equations [20, 24, 26]. Wang et al. [21, 23] discussed trigonometric collocation methods for multi-frequency oscillatory equations. In [22], Li et al. proposed functionally fitted energy-preserving methods to oscillatory Hamiltonian systems. ERKN integrators is investigated by Wang et al. [25] and the convergence is obtained for multi-frequency oscillatory systems. Wu et al. also presented exponentially fitted modified Runge-Kutta-Nyström methods in [27]. Berghe et al. [28] studied symplectic exponentially fitted Runge-Kutta methods of the Gauss type.

However, the investigation of multisymplectic scheme for oscillatory solutions of Maxwell equations is extremely sparse. In this paper, we apply exponentially fitted trapezoidal scheme to construct multisymplectic scheme to simulate Maxwell equations with oscillatory solutions. Our main contributions are highlighted as follows:

- We adopt exponentially fitted method to establish a multisymplectic scheme for solving Maxwell equations with periodic solutions.
- To reduce the calculations, we present a LOD multisymplectic scheme by means of splitting method.
- We rigorously prove the conservation, stability, dispersion and convergence theorems of our method.
- Numerical experiment of Maxwell equations verifies the effectiveness of the LOD multisymplectic scheme in simulating high-frequency oscillatory equations. Meanwhile numerical inversion results show that least square method and LOD multisymplectic scheme can be combined to fit the electric permittivity under small measured errors.

The rest of this paper is outlined as follows. In section 2, we commence by reviewing some preliminary knowledge of Maxwell equations and recall exponentially fitted trapezoidal scheme. In section 3, a new multisymplectic scheme is proposed by utilising exponentially fitted trapezoidal scheme and we proceed with the proof of multisymplectic conservation law and two energy conservation laws. Then we built a splitting multisymplectic scheme by splitting original equations into three LOD systems in section 4. We also analyze its numerical stability, dispersion relation and convergence. In section 5, we present two numerical examples to verify the theoretical analysis and confirm the effectiveness of our method. Numerical test is also given on fitting the electric permittivity by applying the least square method and exponentially fitted trapezoidal scheme.

## Preliminaries

Maxwell equations are the theoretical basis of electrodynamics. Maxwell equations are equivalent to multisymplectic Hamiltonian systems. From the multisymplectic form, we can construct multisymplectic schemes of Maxwell equations.

### Multisymplectic structure of Maxwell equations

For isotropic homogeneous medium, by assuming that there is no free charge and conduction current, the propagation law of electromagnetic field can be described by Maxwell’s equations in the following form of curl

$$\begin{array}{c} \left\{ \begin{array}{cc} & \frac{\partial E}{\partial t}=\frac{1}{\varepsilon }\nabla \times H,\\ & \frac{\partial H}{\partial t}=-\frac{1}{\mu }\nabla \times E. \end{array}\right. \end{array}$$ (1)

Here *E* = (*E*_*x*_, *E*_*y*_, *E*_*z*_)^*T*^ is electric field and *H* = (*H*_*x*_, *H*_*y*_, *H*_*z*_)^*T*^ is magnetic field. *ε* and *μ* denote permittivity and permeability, respectively. Below *ε* and *μ* are constants. Assume that *t* ∈ [0, *T*], (*x*, *y*, *z*) ∈ Ω = [0, *a*] × [0, *b*] × [0, *c*]. The corresponding component form of the system (1) is as follows

$$\begin{array}{c} \left\{ \begin{array}{cc} & \frac{\partial E_{x}}{\partial t}=\frac{1}{\varepsilon }(\frac{\partial H_{z}}{\partial y}-\frac{\partial H_{y}}{\partial z}),\\ & \frac{\partial E_{y}}{\partial t}=\frac{1}{\varepsilon }(\frac{\partial H_{x}}{\partial z}-\frac{\partial H_{z}}{\partial x}),\\ & \frac{\partial E_{z}}{\partial t}=\frac{1}{\varepsilon }(\frac{\partial H_{y}}{\partial x}-\frac{\partial H_{x}}{\partial y}),\\ & \frac{\partial H_{x}}{\partial t}=-\frac{1}{\mu }(\frac{\partial E_{z}}{\partial y}-\frac{\partial E_{y}}{\partial z}),\\ & \frac{\partial H_{y}}{\partial t}=-\frac{1}{\mu }(\frac{\partial E_{x}}{\partial z}-\frac{\partial E_{z}}{\partial x}),\\ & \frac{\partial H_{z}}{\partial t}=-\frac{1}{\mu }(\frac{\partial E_{y}}{\partial x}-\frac{\partial E_{x}}{\partial y}). \end{array}\right. \end{array}$$ (2)

By introducing a new variable *Z* = (*H*_*x*_, *H*_*y*_, *H*_*z*_, *E*_*x*_, *E*_*y*_, *E*_*z*_)^*T*^, the system (2) is equivalent to the following multisymplectic Hamiltonian system

$$\begin{array}{c} M\frac{\partial Z}{\partial t}+K_{1}\frac{\partial Z}{\partial x}+K_{2}\frac{\partial Z}{\partial y}+K_{3}\frac{\partial Z}{\partial z}=\nabla_{Z}S\left(Z\right), \end{array}$$ (3)

Here the Hamiltonian function is *S*(*Z*) = 0, and corresponding antisymmetric matrices are

$$\begin{array}{c} M=[\begin{array}{cc} 0_{3} & -I_{3}\\ I_{3} & 0_{3} \end{array}],K_{1}=[\begin{array}{cc} \frac{1}{\varepsilon }A_{1} & 0_{3}\\ 0_{3} & \frac{1}{\mu }A_{1} \end{array}],K_{2}=[\begin{array}{cc} \frac{1}{\varepsilon }A_{2} & 0_{3}\\ 0_{3} & \frac{1}{\mu }A_{2} \end{array}],K_{3}=[\begin{array}{cc} \frac{1}{\varepsilon }A_{3} & 0_{3}\\ 0_{3} & \frac{1}{\mu }A_{3} \end{array}], \end{array}$$

$$\begin{array}{c} A_{1}=[\begin{array}{ccc} 0 & 0 & 0\\ 0 & 0 & -1\\ 0 & 1 & 0 \end{array}],A_{2}=[\begin{array}{ccc} 0 & 0 & 1\\ 0 & 0 & 0\\ -1 & 0 & 0 \end{array}],A_{3}=[\begin{array}{ccc} 0 & -1 & 0\\ 1 & 0 & 0\\ 0 & 0 & 0 \end{array}], \end{array}$$

where **0**_3_ and *I*_3_ denote third order zero square matrix and unit square matrix, respectively. Multisymplectic system (3) satisfies the following multisymplectic conservation law

$$\begin{array}{c} \frac{\partial }{\partial t}\omega +\frac{\partial }{\partial x}\kappa_{1}+\frac{\partial }{\partial y}\kappa_{2}+\frac{\partial }{\partial z}\kappa_{3}=0, \end{array}$$ (4)

where

$$\begin{array}{cc} & \omega =\frac{1}{2}dZ\wedge MdZ=dE_{x}\wedge dH_{x}+dE_{y}\wedge dH_{y}+dE_{z}\wedge dH_{z},\\ & \kappa_{1}=\frac{1}{2}dZ\wedge K_{1}dZ=\frac{1}{\varepsilon }dH_{z}\wedge dH_{y}+\frac{1}{\mu }dE_{z}\wedge dE_{y},\\ & \kappa_{2}=\frac{1}{2}dZ\wedge K_{2}dZ=\frac{1}{\varepsilon }dH_{x}\wedge dH_{z}+\frac{1}{\mu }dE_{x}\wedge dE_{z},\\ & \kappa_{3}=\frac{1}{2}dZ\wedge K_{3}dZ=\frac{1}{\varepsilon }dH_{y}\wedge dH_{x}+\frac{1}{\mu }dE_{y}\wedge dE_{x}. \end{array}$$

Under periodic boundary condition, Maxwell equations have two energy invariants as follows:

$$\begin{array}{cc} I_{1} & =\int_{\Omega }{\varepsilon |E\left(x,t\right)|}^{2}+\mu {\left|H\left(x,t\right)\right|}^{2}d\Omega , \end{array}$$ (5)

$$\begin{array}{cc} I_{2} & =\int_{\Omega }\varepsilon {\left|\frac{\partial E(x,t)}{\partial t}\right|}^{2}+\mu {\left|\frac{\partial H(x,t)}{\partial t}\right|}^{2}d\Omega . \end{array}$$ (6)

In the case of one-dimensional transverse magnetic polarization, the electric field is *E* = (0, 0, *E*_*z*_)^*T*^, and the magnetic field is *H* = (0, *H*_*y*_, 0)^*T*^. Then the transmission law of electromagnetic field accords with the following one-dimensional Maxwell equations

$$\begin{array}{c} \left\{ \begin{array}{cc} & \frac{\partial E_{z}}{\partial t}=\frac{1}{\varepsilon }\frac{\partial H_{y}}{\partial x},\\ & \frac{\partial H_{y}}{\partial t}=\frac{1}{\mu }\frac{\partial E_{z}}{\partial x}. \end{array}\right. \end{array}$$ (7)

### Exponentially fitted trapezoidal scheme

The exponentially fitted Runge-Kutta method is a kind of efficient numerical tool to simulate ordinary differential equation with oscillatory solutions [27–30]. For example, exponentially fitted trapezoidal scheme is a special exponentially fitted Runge-Kutta method. Yin et al. [30] applied exponentially fitted trapezoidal scheme to simulate a kind of stochastic oscillator system and obtained efficient and stable numerical results.

For initial value problem of ordinary differential equation *u*′ = *f*(*t*, *u*), *u*(*t*_0_) = *u*_0_, exponentially fitted trapezoidal scheme has the following formula

$$\begin{array}{c} \begin{array}{c} u_{1}=u_{0}+\frac{1-\cos(\omega_{t}\Delta t)}{\omega_{t}\sin\left(\omega_{t}\Delta t\right)}\left(f\left(t_{0},u_{0}\right)+f\left(t_{1},u_{1}\right)\right), \end{array} \end{array}$$ (8)

where *ω*_*t*_ denotes the frequency parameter of the scheme [31]. By Taylor expansion, the local truncation error of the method is $\left(u^{\left(3\right)}+\omega_{t}^{2}u^{\prime }\right)\Delta t^{3}/12.$ Therefore, exponentially fitted trapezoidal scheme is convergent with second order accuracy. If we choose *ω*_*t*_ such that $u^{\left(3\right)}+\omega_{t}^{2}u^{\prime }=0,$ third order convergence is obtained.

## Exponentially fitted multisymplectic scheme of Maxwell equations

Based on the multisymplectic formula (3), we can design multisymplectic schemes of Maxwell equations with oscillatory solutions by applying exponentially fitted Runge-Kutta method. Below, we take exponentially fitted trapezoidal scheme as an example.

### Derivation of exponentially fitted multisymplectic scheme

In temporal and spatial discretization of multisymplectic Hamiltonian system (3), we apply exponentially fitted trapezoidal scheme.

First, divide the solution area into equidistant meshes. The step sizes taken in the directions of time and space are denoted by Δ*t*, Δ*x*, Δ*y*, Δ*z*, respectively. Denote the mesh points in the directions of time and space by

$$t_{n}=n\Delta t,\;x_{i}=i\Delta x,\;y_{j}=j\Delta y,\;z_{k}=k\Delta z,$$

respectively, where *n* = 0, 1, 2, ⋯, *N*_*t*_, *i* = 0, 1, 2, ⋯, *N*_*x*_, *j* = 0, 1, 2, ⋯, *N*_*y*_, *k* = 0, 1, 2, ⋯, *N*_*z*_,

$$N_{t}=T/\Delta t,N_{x}=a/\Delta x,N_{y}=b/\Delta y,N_{z}=c/\Delta z.$$

The numerical solutions of *u*(*t*, *x*, *y*, *z*) at points

$$\left(t_{n},x_{i},y_{j},z_{k}\right),\;\left(t_{n},x,y,z\right),\;\left(t,x_{i},y,z\right),\;\left(t,x,y_{j},z\right),\;\left(t,x,y,z_{k}\right)$$

are denoted by $u_{i,j,k}^{n},\;u^{n},\;u_{i},\;u_{j},\;u_{k},$ respectively. We also make the following notions:

$$u^{n+\frac{1}{2}}=\frac{u^{n}+u^{n+1}}{2},\;u_{i+\frac{1}{2}}=\frac{u_{i}+u_{i+1}}{2},\;u_{j+\frac{1}{2}}=\frac{u_{j}+u_{j+1}}{2},\;u_{k+\frac{1}{2}}=\frac{u_{k}+u_{k+1}}{2}.$$

Define the difference operator as follows

$$\begin{array}{c} \delta_{t}u^{n}=u^{n+1}-u^{n},\;\delta_{x}u_{i}=u_{i+1}-u_{i},\;\delta_{y}u_{j}=u_{j+1}-u_{j},\;\delta_{z}u_{k}=u_{k+1}-u_{k}. \end{array}$$

Below, we apply exponentially fitted trapezoidal scheme in temporal and spatial directions to discretize the multisymplectic Hamiltonian system (3) and get the numerical method as follows:

$$\begin{array}{c} M\frac{\delta_{t}Z_{i+\frac{1}{2},j+\frac{1}{2},k+\frac{1}{2}}^{n}}{\alpha_{t}}+K_{1}\frac{\delta_{x}Z_{i,j+\frac{1}{2},k+\frac{1}{2}}^{n+\frac{1}{2}}}{\alpha_{x}}+K_{2}\frac{\delta_{y}Z_{i+\frac{1}{2},j,k+\frac{1}{2}}^{n+\frac{1}{2}}}{\alpha_{y}}+K_{3}\frac{\delta_{z}Z_{i+\frac{1}{2},j+\frac{1}{2},k}^{n+\frac{1}{2}}}{\alpha_{z}}=0. \end{array}$$

For convenience, we give the following simple notions in above formula:

$$\alpha_{t}=\frac{1-\cos(\omega_{t}\Delta t)}{\omega_{t}\sin\left(\omega_{t}\Delta t\right)},\alpha_{x}=\frac{1-\cos(\omega_{x}\Delta x)}{\omega_{x}\sin\left(\omega_{x}\Delta x\right)},\alpha_{y}=\frac{1-\cos(\omega_{y}\Delta y)}{\omega_{y}\sin\left(\omega_{y}\Delta y\right)},\alpha_{z}=\frac{1-\cos(\omega_{z}\Delta z)}{\omega_{z}\sin\left(\omega_{z}\Delta z\right)},$$

where *ω*_*t*_, *ω*_*x*_, *ω*_*y*_, *ω*_*z*_ are the frequency parameters of exponentially schemes applied in the direction of time and space, respectively.

For the sake of simplicity, below we omit the semi-node indices. The omitted indices in below are the semi-node indices. We abbreviate above formula as

$$\begin{array}{c} M\frac{\delta_{t}Z^{n}}{\alpha_{t}}+K_{1}\frac{\delta_{x}Z_{i}}{\alpha_{x}}+K_{2}\frac{\delta_{y}Z_{j}}{\alpha_{y}}+K_{3}\frac{\delta_{z}Z_{k}}{\alpha_{z}}=0. \end{array}$$ (9)

The corresponding component form of above scheme (9) is

$$\begin{array}{c} \left\{ \begin{array}{cc} & \frac{\delta_{t}E_{x}^{n}}{\alpha_{t}}=\frac{1}{\varepsilon }(\frac{\delta_{y}H_{zj}}{\alpha_{y}}-\frac{\delta_{z}H_{yk}}{\alpha_{z}}),\\ & \frac{\delta_{t}E_{y}^{n}}{\alpha_{t}}=\frac{1}{\varepsilon }(\frac{\delta_{z}H_{xk}}{\alpha_{z}}-\frac{\delta_{x}H_{zi}}{\alpha_{x}}),\\ & \frac{\delta_{t}E_{z}^{n}}{\alpha_{t}}=\frac{1}{\varepsilon }(\frac{\delta_{x}H_{yi}}{\alpha_{x}}-\frac{\delta_{y}H_{xj}}{\alpha_{y}}),\\ & \frac{\delta_{t}H_{x}^{n}}{\alpha_{t}}=-\frac{1}{\mu }(\frac{\delta_{y}E_{zj}}{\alpha_{y}}-\frac{\delta_{z}E_{yk}}{\alpha_{z}}),\\ & \frac{\delta_{t}H_{y}^{n}}{\alpha_{t}}=-\frac{1}{\mu }(\frac{\delta_{z}E_{xk}}{\alpha_{z}}-\frac{\delta_{x}E_{zi}}{\alpha_{x}}),\\ & \frac{\delta_{t}H_{z}^{n}}{\alpha_{t}}=-\frac{1}{\mu }(\frac{\delta_{x}E_{yi}}{\alpha_{x}}-\frac{\delta_{y}E_{xj}}{\alpha_{y}}). \end{array}\right. \end{array}$$ (10)

### Numerical properties of exponentially fitted multisymplectic scheme

Next we analyze and give numerical properties of our proposed scheme (9).

**Theorem 1** The scheme (9) satisfies the following multisymplectic conservation law

$$\begin{array}{c} \frac{\delta_{t}\omega^{n}}{\alpha_{t}}+\frac{\delta_{x}{\left(\kappa_{1}\right)}_{i}}{\alpha_{x}}+\frac{\delta_{y}{\left(\kappa_{2}\right)}_{j}}{\alpha_{y}}+\frac{\delta_{z}{\left(\kappa_{3}\right)}_{k}}{\alpha_{z}}=0, \end{array}$$ (11)

where $\omega^{n}=\frac{1}{2}dZ^{n}\wedge MdZ^{n},{\left(\kappa_{1}\right)}_{i}=\frac{1}{2}dZ_{i}\wedge K_{1}dZ_{i},{\left(\kappa_{2}\right)}_{j}=\frac{1}{2}dZ_{j}\wedge K_{2}dZ_{j},{\left(\kappa_{3}\right)}_{k}=\frac{1}{2}dZ_{k}\wedge K_{3}dZ_{k}.$ Here for the sake of simplicity, we omit the semi-node indices. So the scheme (9) is a multisymplectic scheme of Maxwell equations.

**Proof** Taking variation on both sides of the formula (9) yields that

$$\begin{array}{c} M\frac{\delta_{t}dZ^{n}}{\alpha_{t}}+K_{1}\frac{\delta_{x}dZ_{i}}{\alpha_{x}}+K_{2}\frac{\delta_{y}dZ_{j}}{\alpha_{y}}+K_{3}\frac{\delta_{z}dZ_{k}}{\alpha_{z}}=0. \end{array}$$

Then by making wedge product on both sides of above formula with $dZ^{n+\frac{1}{2}}$ and considering the properties of antisymmetric matrix and wedge product, we obtain the discrete multisymplectic conservation law (11).

Below, we refer to the scheme (9) as exponentially fitted multisymplectic (abbreviated to EFMS) scheme.

**Theorem 2** Under periodic boundary condition, EFMS scheme (9) has two discerete energy invariants as follows:

1.
$I_{1}^{n}=\varepsilon \|E^{n}{\|}^{2}+\mu {\left\|H^{n}\right\|}^{2},$

2.
$I_{2}^{n}=\varepsilon \|\delta_{t}E^{n}{\|}^{2}+\mu {\left\|\delta_{t}H^{n}\right\|}^{2},$

where

$$\begin{array}{cc} \|E^{n}{\|}^{2} & =\alpha_{x}\alpha_{y}\alpha_{z}\sum_{i=1}^{N_{x}}\sum_{j=1}^{N_{y}}\sum_{k=1}^{N_{z}}[{\left(E_{x}^{n}\right)}^{2}+{\left(E_{y}^{n}\right)}^{2}+{\left(E_{z}^{n}\right)}^{2}], \end{array}$$

$$\begin{array}{cc} \|\delta_{t}H^{n}{\|}^{2} & =\alpha_{x}\alpha_{y}\alpha_{z}\sum_{i=1}^{N_{x}}\sum_{j=1}^{N_{y}}\sum_{k=1}^{N_{z}}[{\left(\delta_{t}H_{x}^{n}\right)}^{2}+{\left(\delta_{t}H_{y}^{n}\right)}^{2}+{\left(\delta_{t}H_{z}^{n}\right)}^{2}], \end{array}$$

and other two norms have similar definitions. Here for the sake of simplicity, we omit the semi-node indices.

**Proof** By taking inner product on both sides of EFMS scheme (9) with $Z^{n+\frac{1}{2}}$ we obtain that

$$\begin{array}{c} M\frac{\left(\delta_{t}Z^{n},Z^{n+\frac{1}{2}}\right)}{\alpha_{t}}+K_{1}\frac{\left(\delta_{x}Z_{i},Z^{n+\frac{1}{2}}\right)}{\alpha_{x}}+K_{2}\frac{\left(\delta_{y}Z_{j},Z^{n+\frac{1}{2}}\right)}{\alpha_{y}}+K_{3}\frac{\left(\delta_{z}Z_{k},Z^{n+\frac{1}{2}}\right)}{\alpha_{z}}=0. \end{array}$$ (12)

Above formula can be rewritten as the component form as follows

$$\begin{array}{c} \left\{ \begin{array}{cc} & \varepsilon \frac{\delta_{t}E_{x}^{n}E_{x}^{n+\frac{1}{2}}}{\alpha_{t}}=\frac{\delta_{y}H_{zj}E_{x}^{n+\frac{1}{2}}}{\alpha_{y}}-\frac{\delta_{z}H_{yk}E_{x}^{n+\frac{1}{2}}}{\alpha_{z}},\\ & \varepsilon \frac{\delta_{t}E_{y}^{n}E_{y}^{n+\frac{1}{2}}}{\alpha_{t}}=\frac{\delta_{z}H_{xk}E_{y}^{n+\frac{1}{2}}}{\alpha_{z}}-\frac{\delta_{x}H_{zi}E_{y}^{n+\frac{1}{2}}}{\alpha_{x}},\\ & \varepsilon \frac{\delta_{t}E_{z}^{n}E_{z}^{n+\frac{1}{2}}}{\alpha_{t}}=\frac{\delta_{x}H_{yi}E_{z}^{n+\frac{1}{2}}}{\alpha_{x}}-\frac{\delta_{y}H_{xj}E_{z}^{n+\frac{1}{2}}}{\alpha_{y}},\\ & \mu \frac{\delta_{t}H_{x}^{n}H_{x}^{n+\frac{1}{2}}}{\alpha_{t}}=-\frac{\delta_{y}E_{zj}H_{x}^{n+\frac{1}{2}}}{\alpha_{y}}+\frac{\delta_{z}E_{yk}H_{x}^{n+\frac{1}{2}}}{\alpha_{z}},\\ & \mu \frac{\delta_{t}H_{y}^{n}H_{y}^{n+\frac{1}{2}}}{\alpha_{t}}=-\frac{\delta_{z}E_{xk}H_{y}^{n+\frac{1}{2}}}{\alpha_{z}}+\frac{\delta_{x}E_{zi}H_{y}^{n+\frac{1}{2}}}{\alpha_{x}},\\ & \mu \frac{\delta_{t}H_{z}^{n}H_{z}^{n+\frac{1}{2}}}{\alpha_{t}}=-\frac{\delta_{x}E_{yi}H_{z}^{n+\frac{1}{2}}}{\alpha_{x}}+\frac{\delta_{y}E_{xj}H_{z}^{n+\frac{1}{2}}}{\alpha_{y}}, \end{array}\right. \end{array}$$ (13)

where the omitted indices are semi-nods indices. Obviously

$$\delta_{t}E_{x}^{n}E_{x}^{n+\frac{1}{2}}=\frac{1}{2}[{\left(E_{x}^{n+1}\right)}^{2}-{\left(E_{x}^{n}\right)}^{2}].$$

In view of periodic boundary condition, we get that

$$\sum_{j=1}^{N_{y}}(\delta_{y}H_{zj}E_{x}^{n+\frac{1}{2}}+\delta_{y}E_{xj}H_{z}^{n+\frac{1}{2}})=0,$$

and other five similar equalities. Therefore, summing all terms on both sides of above equation (13) with respect to the indices *i*, *j*, *k* yields the first discrete energy conservation law $I_{1}^{n}=I_{1}^{n+1}.$

By applying difference operator *δ*_*t*_ to EFMS scheme (9), we obtain that

$$\begin{array}{c} M\frac{\delta_{t}Z^{n+1}-\delta_{t}Z^{n}}{\alpha_{t}}+K_{1}\frac{\delta_{t}\delta_{x}Z_{i}}{\alpha_{x}}+K_{2}\frac{\delta_{t}\delta_{y}Z_{j}}{\alpha_{y}}+K_{3}\frac{\delta_{t}\delta_{z}Z_{k}}{\alpha_{z}}=0. \end{array}$$

Making inner product on both sides of above formula with $\delta_{t}Z^{n+\frac{1}{2}}$ yields that

$$\begin{array}{cc} & M\frac{\left(\delta_{t}Z^{n+1}-\delta_{t}Z^{n},\delta_{t}Z^{n+\frac{1}{2}}\right)}{\alpha_{t}}+K_{1}\frac{\left(\delta_{t}\delta_{x}Z_{i},\delta_{t}Z^{n+\frac{1}{2}}\right)}{\alpha_{x}}\\ & \;+K_{2}\frac{\left(\delta_{t}\delta_{y}Z_{j},\delta_{t}Z^{n+\frac{1}{2}}\right)}{\alpha_{y}}+K_{3}\frac{\left(\delta_{t}\delta_{z}Z_{k},\delta_{t}Z^{n+\frac{1}{2}}\right)}{\alpha_{z}}=0. \end{array}$$

Its equivalent componentwise form is

$$\begin{array}{c} \left\{ \begin{array}{cc} & \varepsilon \frac{\left(\delta_{t}E_{x}^{n+1}-\delta_{t}E_{x}^{n}\right)\delta_{t}E_{x}^{n+\frac{1}{2}}}{\alpha_{t}}=\frac{\delta_{t}\delta_{y}H_{zj}\delta_{t}E_{x}^{n+\frac{1}{2}}}{\alpha_{y}}-\frac{\delta_{t}\delta_{z}H_{yk}\delta_{t}E_{x}^{n+\frac{1}{2}}}{\alpha_{z}},\\ & \varepsilon \frac{\left(\delta_{t}E_{y}^{n+1}-\delta_{t}E_{y}^{n}\right)\delta_{t}E_{y}^{n+\frac{1}{2}}}{\alpha_{t}}=\frac{\delta_{t}\delta_{z}H_{xk}\delta_{t}E_{y}^{n+\frac{1}{2}}}{\alpha_{z}}-\frac{\delta_{t}\delta_{x}H_{zi}\delta_{t}E_{y}^{n+\frac{1}{2}}}{\alpha_{x}},\\ & \varepsilon \frac{\left(\delta_{t}E_{z}^{n+1}-\delta_{t}E_{z}^{n}\right)\delta_{t}E_{z}^{n+\frac{1}{2}}}{\alpha_{t}}=\frac{\delta_{t}\delta_{x}H_{yi}\delta_{t}E_{z}^{n+\frac{1}{2}}}{\alpha_{x}}-\frac{\delta_{t}\delta_{y}H_{xj}\delta_{t}E_{z}^{n+\frac{1}{2}}}{\alpha_{y}},\\ & \mu \frac{\left(\delta_{t}H_{x}^{n+1}-\delta_{t}H_{x}^{n}\right)\delta_{t}H_{x}^{n+\frac{1}{2}}}{\alpha_{t}}=-\frac{\delta_{t}\delta_{y}E_{zj}\delta_{t}H_{x}^{n+\frac{1}{2}}}{\alpha_{y}}+\frac{\delta_{t}\delta_{z}E_{yk}\delta_{t}H_{x}^{n+\frac{1}{2}}}{\alpha_{z}},\\ & \mu \frac{\left(\delta_{t}H_{y}^{n+1}-\delta_{t}H_{y}^{n}\right)\delta_{t}H_{y}^{n+\frac{1}{2}}}{\alpha_{t}}=-\frac{\delta_{t}\delta_{z}E_{xk}\delta_{t}H_{y}^{n+\frac{1}{2}}}{\alpha_{z}}+\frac{\delta_{t}\delta_{x}E_{zi}\delta_{t}H_{y}^{n+\frac{1}{2}}}{\alpha_{x}},\\ & \mu \frac{\left(\delta_{t}H_{z}^{n+1}-\delta_{t}H_{z}^{n}\right)\delta_{t}H_{z}^{n+\frac{1}{2}}}{\alpha_{t}}=-\frac{\delta_{t}\delta_{x}E_{yi}\delta_{t}H_{z}^{n+\frac{1}{2}}}{\alpha_{x}}+\frac{\delta_{t}\delta_{y}E_{xj}\delta_{t}H_{z}^{n+\frac{1}{2}}}{\alpha_{y}}, \end{array}\right. \end{array}$$ (14)

where the omitted indices are semi-nods indices. Clearly,

$$\left(\delta_{t}E_{x}^{n+1}-\delta_{t}E_{x}^{n}\right)\delta_{t}E_{x}^{n+\frac{1}{2}}=\frac{1}{2}[{\left(\delta_{t}E_{x}^{n+1}\right)}^{2}-{\left(\delta_{t}E_{x}^{n}\right)}^{2}].$$

On account of periodic boundary condition and commutativity of difference operators, we derive that

$$\sum_{i=1}^{N_{x}}(\delta_{t}\delta_{x}H_{zi}\delta_{t}E_{y}^{n+\frac{1}{2}}+\delta_{t}\delta_{x}E_{yi}\delta_{t}H_{z}^{n+\frac{1}{2}})=0,$$

and other five similar equalities. Hence, by summing all terms on both sides of above equation (14) with respect to the indices *i*, *j*, *k*, we obtain the second discrete energy conservation law $I_{2}^{n}=I_{2}^{n+1}.$

As is well known that if the media is lossless, the system (1) is divergence-free [5, 6], i.e.,

$$\begin{array}{c} \text{div}(E(t))=\text{div}(E(0)),\;\text{div}(H(t))=\text{div}(H(0)). \end{array}$$ (15)

The divergence convergence is satisfied by EFMS scheme.

**Theorem 3** EFMS scheme (9) preserves the following two discrete divergences:

$$\begin{array}{c} {\bar{\nabla }}_{i,j,k}\cdot E^{n+1}={\bar{\nabla }}_{i,j,k}\cdot E^{n},\;{\bar{\nabla }}_{i,j,k}\cdot H^{n+1}={\bar{\nabla }}_{i,j,k}\cdot H^{n}. \end{array}$$ (16)

Here the discrete divergence operator is defined by

$$\begin{array}{c} {\bar{\nabla }}_{i,j,k}\cdot (\begin{array}{c} X\\ Y\\ Z \end{array})=\frac{\delta_{x}}{\alpha_{x}}X_{i-\frac{1}{2},\bar{j},\bar{k}}+\frac{\delta_{y}}{\alpha_{y}}Y_{\bar{i},j-\frac{1}{2},\bar{k}}+\frac{\delta_{z}}{\alpha_{z}}Z_{\bar{i},\bar{j},k-\frac{1}{2}}, \end{array}$$

where

$$X_{i-\frac{1}{2},\bar{j},\bar{k}}=X_{i-\frac{1}{2},j+\frac{1}{2},k+\frac{1}{2}}+X_{i-\frac{1}{2},j+\frac{1}{2},k-\frac{1}{2}}+X_{i-\frac{1}{2},j-\frac{1}{2},k+\frac{1}{2}}+X_{i-\frac{1}{2},j-\frac{1}{2},k-\frac{1}{2}},$$

and other symbols are similar defined. (16) can be seen as the discrete conservation law of (15).

**Proof** Above operator definition yields that

$$\begin{array}{ccc} & & {\bar{\nabla }}_{i,j,k}\cdot E^{n+1}-{\bar{\nabla }}_{i,j,k}\cdot E^{n}\\ & & =\frac{\delta_{x}}{\alpha_{x}}\delta_{t}{\left(E_{x}\right)}_{i-\frac{1}{2},\bar{j},\bar{k}}^{n}+\frac{\delta_{y}}{\alpha_{y}}\delta_{t}{\left(E_{y}\right)}_{\bar{i},j-\frac{1}{2},\bar{k}}^{n}+\frac{\delta_{z}}{\alpha_{z}}\delta_{t}{\left(E_{z}\right)}_{\bar{i},\bar{j},k-\frac{1}{2}}^{n}. \end{array}$$

According to formula (10) of EFMS scheme, we obtain that

$$\begin{array}{ccc} & & {\bar{\nabla }}_{i,j,k}\cdot E^{n+1}-{\bar{\nabla }}_{i,j,k}\cdot E^{n}\\ & & =\frac{\alpha_{t}\delta_{x}}{\varepsilon \alpha_{x}}(\frac{\delta_{y}{\left(H_{z}\right)}_{i-\frac{1}{2},\hat{j},\bar{k}}}{\alpha_{y}}-\frac{\delta_{z}{\left(H_{y}\right)}_{i-\frac{1}{2},\bar{j},\hat{k}}}{\alpha_{z}})\\ & & \;+\frac{\alpha_{t}\delta_{y}}{\varepsilon \alpha_{y}}(\frac{\delta_{z}{\left(H_{x}\right)}_{\bar{i},j-\frac{1}{2},\hat{k}}}{\alpha_{z}}-\frac{\delta_{x}{\left(H_{z}\right)}_{\hat{i},j-\frac{1}{2},\bar{k}}}{\alpha_{x}})\\ & & \;+\frac{\alpha_{t}\delta_{z}}{\varepsilon \alpha_{z}}(\frac{\delta_{x}{\left(H_{y}\right)}_{\hat{i},\bar{j},k-\frac{1}{2}}}{\alpha_{x}}-\frac{\delta_{y}{\left(H_{x}\right)}_{\bar{i},\hat{j},k-\frac{1}{2}}}{\alpha_{y}}), \end{array}$$

where

$$X_{i-\frac{1}{2},\hat{j},\bar{k}}=X_{i-\frac{1}{2},j,k+\frac{1}{2}}+X_{i-\frac{1}{2},j,k-\frac{1}{2}}+X_{i-\frac{1}{2},j-1,k+\frac{1}{2}}+X_{i-\frac{1}{2},j-1,k-\frac{1}{2}},$$

and other symbols are similar defined. By calculation we can derive

$$\delta_{x}\delta_{y}{\left(H_{z}\right)}_{i-\frac{1}{2},\hat{j},\bar{k}}=\delta_{y}\delta_{x}{\left(H_{z}\right)}_{\hat{i},j-\frac{1}{2},\bar{k}}$$

and other two similar equalities, which completes the proof of the first divergence convergence. The proof of the second assertion is similar.

## LOD exponentially fitted multisymplectic scheme

According to the splitting method of vector field [32–34], to reduce calculation cost, we consider the following LOD system of multisymplectic Hamiltonian system (3)

$$\begin{array}{c} \frac{1}{3}MZ_{t}+K_{1}Z_{x}=0,\;\frac{1}{3}MZ_{t}+K_{2}Z_{y}=0,\;\frac{1}{3}MZ_{t}+K_{3}Z_{z}=0. \end{array}$$ (17)

The system (17) has LOD multisymplectic conservation law

$$\begin{array}{c} \frac{1}{3}\frac{\partial }{\partial t}\omega +\frac{\partial }{\partial x}\kappa_{x}=0,\;\frac{1}{3}\frac{\partial }{\partial t}\omega +\frac{\partial }{\partial y}\kappa_{y}=0,\;\frac{1}{3}\frac{\partial }{\partial t}\omega +\frac{\partial }{\partial z}\kappa_{z}=0. \end{array}$$ (18)

We recast the system (17) into the following six one-dimensional Maxwell equations:

$$\begin{array}{c} \left\{ \begin{array}{cc} & \frac{\partial E_{z}}{\partial t}=\frac{3}{\varepsilon }\frac{\partial H_{y}}{\partial x},\\ & \frac{\partial H_{y}}{\partial t}=\frac{3}{\mu }\frac{\partial E_{z}}{\partial x}, \end{array}\{\begin{array}{cc} & \frac{\partial E_{y}}{\partial t}=-\frac{3}{\varepsilon }\frac{\partial H_{z}}{\partial x},\\ & \frac{\partial H_{z}}{\partial t}=-\frac{3}{\mu }\frac{\partial E_{y}}{\partial x}, \end{array}\right. \end{array}$$ (19)

$$\begin{array}{c} \left\{ \begin{array}{cc} & \frac{\partial E_{x}}{\partial t}=\frac{3}{\varepsilon }\frac{\partial H_{z}}{\partial y},\\ & \frac{\partial H_{z}}{\partial t}=\frac{3}{\mu }\frac{\partial E_{x}}{\partial y}, \end{array}\{\begin{array}{cc} & \frac{\partial E_{z}}{\partial t}=-\frac{3}{\varepsilon }\frac{\partial H_{x}}{\partial y},\\ & \frac{\partial H_{x}}{\partial t}=-\frac{3}{\mu }\frac{\partial E_{z}}{\partial y}, \end{array}\right. \end{array}$$ (20)

$$\begin{array}{c} \left\{ \begin{array}{cc} & \frac{\partial E_{y}}{\partial t}=\frac{3}{\varepsilon }\frac{\partial H_{x}}{\partial z},\\ & \frac{\partial H_{x}}{\partial t}=\frac{3}{\mu }\frac{\partial E_{y}}{\partial z}, \end{array}\{\begin{array}{cc} & \frac{\partial E_{x}}{\partial t}=-\frac{3}{\varepsilon }\frac{\partial H_{y}}{\partial z},\\ & \frac{\partial H_{y}}{\partial t}=-\frac{3}{\mu }\frac{\partial E_{x}}{\partial z}. \end{array}\right. \end{array}$$ (21)

Using exponentially fitted trapezoidal scheme to solve numerically the LOD system (17) yields the following LOD scheme

$$\begin{array}{c} \frac{1}{3}M\frac{\delta_{t}Z^{n}}{\alpha_{t}}+K_{1}\frac{\delta_{x}Z_{i}}{\alpha_{x}}=0,\;\frac{1}{3}M\frac{\delta_{t}Z^{n}}{\alpha_{t}}+K_{2}\frac{\delta_{y}Z_{j}}{\alpha_{y}}=0,\;\frac{1}{3}M\frac{\delta_{t}Z^{n}}{\alpha_{t}}+K_{3}\frac{\delta_{z}Z_{k}}{\alpha_{z}}=0. \end{array}$$ (22)

Here for the sake of simplicity, we omit the semi-node indices in above formula. Similarly to Theorem 1, we can prove that above scheme satisfies the discrete LOD multisymplectic conservation law as follows:

$$\begin{array}{c} \frac{1}{3}\frac{\delta_{t}\omega^{n}}{\alpha_{t}}+\frac{\delta_{x}{\left(\kappa_{1}\right)}_{i}}{\alpha_{x}}=0,\;\frac{1}{3}\frac{\delta_{t}\omega^{n}}{\alpha_{t}}+\frac{\delta_{y}{\left(\kappa_{2}\right)}_{j}}{\alpha_{y}}=0,\;\frac{1}{3}\frac{\delta_{t}\omega^{n}}{\alpha_{t}}+\frac{\delta_{z}{\left(\kappa_{3}\right)}_{k}}{\alpha_{z}}=0. \end{array}$$ (23)

So we call the scheme (22) LOD exponentially fitted multisymplectic (abbreviated to LODEFMS) scheme. LODEFMS scheme (22) is equivalent to the following six one-dimensional scheme:

$$\begin{array}{c} \left\{ \begin{array}{cc} & \frac{\delta_{t}E_{z}^{n}}{\alpha_{t}}=\frac{3}{\varepsilon }\frac{\delta_{x}H_{yi}}{\alpha_{x}},\\ & \frac{\delta_{t}H_{y}^{n}}{\alpha_{t}}=\frac{3}{\mu }\frac{\delta_{x}E_{zi}}{\alpha_{x}}, \end{array}\{\begin{array}{cc} & \frac{\delta_{t}E_{y}^{n}}{\alpha_{t}}=-\frac{3}{\varepsilon }\frac{\delta_{x}H_{zi}}{\alpha_{x}},\\ & \frac{\delta_{t}H_{z}^{n}}{\alpha_{t}}=-\frac{3}{\mu }\frac{\delta_{x}E_{yi}}{\alpha_{x}}, \end{array}\right. \end{array}$$ (24)

$$\begin{array}{c} \left\{ \begin{array}{cc} & \frac{\delta_{t}E_{x}^{n}}{\alpha_{t}}=\frac{3}{\varepsilon }\frac{\delta_{y}H_{zj}}{\alpha_{y}},\\ & \frac{\delta_{t}H_{z}^{n}}{\alpha_{t}}=\frac{3}{\mu }\frac{\delta_{y}E_{xj}}{\alpha_{y}}, \end{array}\{\begin{array}{cc} & \frac{\delta_{t}E_{z}^{n}}{\alpha_{t}}=-\frac{3}{\varepsilon }\frac{\delta_{y}H_{xj}}{\alpha_{y}},\\ & \frac{\delta_{t}H_{x}^{n}}{\alpha_{t}}=-\frac{3}{\mu }\frac{\delta_{y}E_{zj}}{\alpha_{y}}, \end{array}\right. \end{array}$$ (25)

$$\begin{array}{c} \left\{ \begin{array}{cc} & \frac{\delta_{t}E_{y}^{n}}{\alpha_{t}}=\frac{3}{\varepsilon }\frac{\delta_{z}H_{xj}}{\alpha_{z}},\\ & \frac{\delta_{t}H_{x}^{n}}{\alpha_{t}}=\frac{3}{\mu }\frac{\delta_{z}E_{yj}}{\alpha_{z}}, \end{array}\{\begin{array}{cc} & \frac{\delta_{t}E_{x}^{n}}{\alpha_{t}}=-\frac{3}{\varepsilon }\frac{\delta_{z}H_{yj}}{\alpha_{z}},\\ & \frac{\delta_{t}H_{y}^{n}}{\alpha_{t}}=-\frac{3}{\mu }\frac{\delta_{z}E_{xj}}{\alpha_{z}}. \end{array}\right. \end{array}$$ (26)

**Theorem 4** LODEFMS scheme (22) is unconditionally stable and non-dissipative scheme.

**Proof** For simplicity, we consider the following one-dimensional Maxwell equations

$$\begin{array}{c} \left\{ \begin{array}{cc} & \frac{\partial E_{y}}{\partial t}+\frac{3}{\varepsilon }\frac{\partial H_{z}}{\partial x}=0,\\ & \frac{\partial H_{z}}{\partial t}+\frac{3}{\mu }\frac{\partial E_{y}}{\partial x}=0, \end{array}\right. \end{array}$$ (27)

and corresponding LODEFMS scheme:

$$\begin{array}{c} \left\{ \begin{array}{cc} & \frac{\delta_{t}E_{y}^{n}}{\alpha_{t}}+\frac{3}{\varepsilon }\frac{\delta_{x}H_{zi}}{\alpha_{x}}=0,\\ & \frac{\delta_{t}H_{z}^{n}}{\alpha_{t}}+\frac{3}{\mu }\frac{\delta_{x}E_{yi}}{\alpha_{x}}=0. \end{array}\right. \end{array}$$ (28)

To analyze the stability of scheme (28), define the following wave solution

$$\begin{array}{c} {}[\begin{array}{c} E_{yi}^{n}\\ H_{zi}^{n} \end{array}]=\rho^{n}\exp^{-\bar{i}\omega_{x}i\Delta_{x}}[\begin{array}{c} E_{y}^{0}\\ H_{z}^{0} \end{array}], \end{array}$$ (29)

where $\bar{i},\omega_{x}$ denote imaginary unit and the wave number, respectively. ${\left[E_{y}^{0},H_{z}^{0}\right]}^{T}$ is a nonzero eigenvector of scheme (28), and *ρ* is the stability factor. Inserting (29) into scheme (28) yields that

$$\begin{array}{c} {}[\begin{array}{cc} (\rho -1)\cos\theta & -\bar{i}\frac{3\alpha_{t}}{\varepsilon \alpha_{x}}\sin\theta \left(\rho +1\right)\\ -\bar{i}\frac{3\alpha_{t}}{\mu \alpha_{x}}\sin\theta \left(\rho +1\right) & (\rho -1)\cos\theta \end{array}][\begin{array}{c} E_{y}^{0}\\ H_{z}^{0} \end{array}]=0, \end{array}$$

where *θ* = *ω*_*x*_Δ_*x*_/2. Making the coefficient determinant 0 leads to

$$\begin{array}{c} \rho^{2}+2\cdot \frac{9\alpha_{t}^{2}tan^{2}\theta -\varepsilon \mu \alpha_{x}^{2}}{9\alpha_{t}^{2}tan^{2}\theta +\varepsilon \mu \alpha_{x}^{2}}\cdot \rho +1=0. \end{array}$$ (30)

Thus, it can be deduced that |*ρ*| = 1. This means that scheme (28) is unconditionally stable and non-dissipative. Similar analysis can prove the stability of other five one-dimensional schemes.

**Theorem 5** Maxwell equations (17) satisfy the following exact dispersive relations

$$\begin{array}{c} \omega_{t}^{2}-\frac{9}{\varepsilon \mu }\omega_{x}^{2}=0,\;\omega_{t}^{2}-\frac{9}{\varepsilon \mu }\omega_{y}^{2}=0,\;\omega_{t}^{2}-\frac{9}{\varepsilon \mu }\omega_{z}^{2}=0. \end{array}$$ (31)

Corresponding numerical dispersive relations of scheme (22) are

$$\begin{array}{c} \frac{1}{\alpha_{t}^{2}}\tan^{2}(\frac{\omega_{t}\Delta_{t}}{2})-\frac{9}{\varepsilon \mu \alpha_{x}^{2}}\tan^{2}(\frac{\omega_{x}\Delta_{x}}{2})=0, \end{array}$$ (32)

$$\begin{array}{c} \frac{1}{\alpha_{t}^{2}}\tan^{2}(\frac{\omega_{t}\Delta_{t}}{2})-\frac{9}{\varepsilon \mu \alpha_{y}^{2}}\tan^{2}(\frac{\omega_{y}\Delta_{y}}{2})=0, \end{array}$$ (33)

$$\begin{array}{c} \frac{1}{\alpha_{t}^{2}}\tan^{2}(\frac{\omega_{t}\Delta_{t}}{2})-\frac{9}{\varepsilon \mu \alpha_{z}^{2}}\tan^{2}(\frac{\omega_{z}\Delta_{z}}{2})=0. \end{array}$$ (34)

Suppose the step sizes tend to 0, numerical dispersive relations converge to exact dispersive relations correspondingly.

**Proof** For the sake of simplicity, take one-dimensional Maxwell equation (27) as an example to analyze dispersion. Set the wave solution of (27) as follows

$$\begin{array}{c} {}[\begin{array}{c} E_{y}\\ H_{z} \end{array}]=\exp^{\bar{i}\left(\omega_{x}x-\omega_{t}t\right)}[\begin{array}{c} E_{y}^{0}\\ H_{z}^{0} \end{array}], \end{array}$$ (35)

were *ω*_*t*_ is the frequency. Substituting (35) into (27) results in

$$\begin{array}{c} {}[\begin{array}{cc} -\omega_{t} & \frac{3}{\varepsilon }\omega_{x}\\ \frac{3}{\mu }\omega_{x} & -\omega_{t} \end{array}][\begin{array}{c} E_{y}^{0}\\ H_{z}^{0} \end{array}]=0. \end{array}$$

Since the eigenvector is nonzero, making above coefficient matrix singular results in the exact dispersive relation

$$\begin{array}{c} \omega_{t}^{2}-\frac{9}{\varepsilon \mu }\omega_{x}^{2}=0. \end{array}$$ (36)

Similar analysis to other five one-dimensional Maxwell equations yields other two exact dispersive relations.

To dispersive relations of scheme (28), inserting the stability factor $\rho =\exp^{\bar{i}\omega_{t}\Delta t}$ into equation (30) leads to

$$\begin{array}{c} \exp^{2\bar{i}\omega_{t}\Delta t}+2\cdot \frac{9\alpha_{t}^{2}tan^{2}\theta -\varepsilon \mu \alpha_{x}^{2}}{9\alpha_{t}^{2}tan^{2}\theta +\varepsilon \mu \alpha_{x}^{2}}\cdot \exp^{\bar{i}\omega_{t}\Delta t}+1=0. \end{array}$$ (37)

In view of the expression of *θ*, we obtain the numerical relation (32). Similarly, we can analyse other five one-dimensional scheme to get other two numerical dispersive relations (33) and (34). Taking the following equivalence relation

$$\lim_{\Delta_{t}\to 0}\frac{\alpha_{t}}{\Delta_{t}}=1,\;\lim_{\Delta_{x}\to 0}\frac{\alpha_{x}}{\Delta_{x}}=1,\;\lim_{\Delta_{t}\to 0}\frac{\alpha_{y}}{\Delta_{y}}=1,\;\lim_{\Delta_{t}\to 0}\frac{\alpha_{z}}{\Delta_{z}}=1$$

into consideration yields that, as the step sizes tend to 0, numerical dispersive relations (32)–(34) converge to the exact relations (36), respectively.

**Fig 1:**
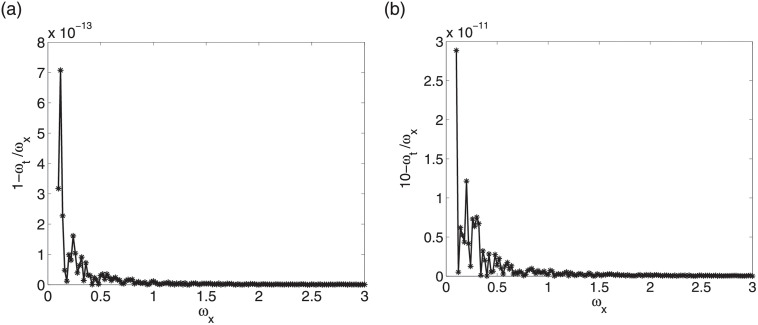
Numerical errors of phase velocity in two data cases.

Fig 1 depicts numerical errors of phase velocity $1/\sqrt{\varepsilon \mu }-\omega_{t}/\omega_{x}.$ The data used in left figure is *ε* = *μ* = 1, Δ_*t*_ = 0.1, Δ_*x*_ = 0.5, while in right figure we use *ε* = *μ* = 0.1, Δ_*t*_ = 0.01, Δ_*x*_ = 0.04. We can see that in the two cases numerical errors of phase velocity are very small. Similar results in other data cases can be obtained.

**Theorem 6** Under precise initial condition, numerical solutions of LODEFMS scheme (22) converge to solutions of Maxwell equations with the following numerical errors:

$$\begin{array}{cc} & \varepsilon \|r^{n+1}{\|}^{2}+\mu {\left\|s^{n+1}\right\|}^{2}\leq \frac{2\alpha_{t}}{2-\alpha_{t}}\exp^{\frac{2n\alpha_{t}}{2-\alpha_{t}}}(\varepsilon \Sigma_{k=1}^{n}\|F^{k+\frac{1}{2}}{\|}^{2}+\mu \Sigma_{k=1}^{n}{\left\|G^{k+\frac{1}{2}}\right\|}^{2}), \end{array}$$ (38)

where *r* and *s* are error vectors of electric field and magnetic field, respectively.

**Proof** For simplicity, we consider errors of one-dimensional scheme to solve one-dimensional Maxwell equation (27). According to local truncation analysis of scheme (28), we obtain that

$$\begin{array}{c} \left\{ \begin{array}{cc} & \frac{\delta_{t}r_{i+\frac{1}{2}}^{n}}{\alpha_{t}}+\frac{3}{\varepsilon }\frac{\delta_{x}s_{i}^{n+\frac{1}{2}}}{\alpha_{x}}=F,\\ & \frac{\delta_{t}s_{i+\frac{1}{2}}^{n}}{\alpha_{t}}+\frac{3}{\mu }\frac{\delta_{x}r_{i}^{n+\frac{1}{2}}}{\alpha_{x}}=G, \end{array}\right. \end{array}$$ (39)

where $r_{i}^{n}=E_{y}\left(x_{i},t^{n}\right)-{\left(E_{y}\right)}_{i}^{n},s_{i}^{n}=H_{z}\left(x_{i},t^{n}\right)-{\left(H_{z}\right)}_{i}^{n},$

$$F=\frac{\Delta t^{2}}{12}(\frac{\partial^{3}E_{y}}{\partial t^{3}}+\omega_{t}^{2}\frac{\partial E_{y}}{\partial t})+\frac{\Delta x^{2}}{12\varepsilon }(\frac{\partial^{3}H_{z}}{\partial x^{3}}+\omega_{x}^{2}\frac{\partial H_{z}}{\partial x}),$$

$$G=\frac{\Delta t^{2}}{12}(\frac{\partial^{3}H_{z}}{\partial t^{3}}+\omega_{t}^{2}\frac{\partial H_{z}}{\partial t})+\frac{\Delta x^{2}}{12\mu }(\frac{\partial^{3}E_{y}}{\partial x^{3}}+\omega_{x}^{2}\frac{\partial E_{y}}{\partial x}).$$

Multiplying the first and second equation of (39) with $r_{i+\frac{1}{2}}^{n+\frac{1}{2}}$ and $s_{i+\frac{1}{2}}^{n+\frac{1}{2}},$ respectively, yields that

$$\begin{array}{c} \left\{ \begin{array}{cc} & \frac{\varepsilon }{\alpha_{t}}[{\left(r_{i+\frac{1}{2}}^{n+1}\right)}^{2}-{\left(r_{i+\frac{1}{2}}^{n}\right)}^{2}]+\frac{3}{\alpha_{x}}\left(s_{i+1}^{n+\frac{1}{2}}-s_{i}^{n+\frac{1}{2}}\right)\left(r_{i+1}^{n+\frac{1}{2}}+r_{i}^{n+\frac{1}{2}}\right)=2\varepsilon Fr_{i+\frac{1}{2}}^{n+\frac{1}{2}},\\ & \frac{\mu }{\alpha_{t}}[{\left(s_{i+\frac{1}{2}}^{n+1}\right)}^{2}-{\left(s_{i+\frac{1}{2}}^{n}\right)}^{2}]+\frac{3}{\alpha_{x}}\left(r_{i+1}^{n+\frac{1}{2}}-r_{i}^{n+\frac{1}{2}}\right)\left(s_{i+1}^{n+\frac{1}{2}}+s_{i}^{n+\frac{1}{2}}\right)=2\mu Gs_{i+\frac{1}{2}}^{n+\frac{1}{2}}. \end{array}\right. \end{array}$$ (40)

By adding both sides of above equation and summing with respect to the subscript *i*, we can get

$$\begin{array}{c} \varepsilon \|r^{n+1}{\|}^{2}+\mu \|s^{n+1}{\|}^{2}=\varepsilon \|r^{n}{\|}^{2}+\mu {\left\|s^{n}\right\|}^{2}+2\varepsilon \alpha_{t}\Sigma_{i}Fr_{i+\frac{1}{2}}^{n+\frac{1}{2}}+2\mu \alpha_{t}\Sigma_{i}Gs_{i+\frac{1}{2}}^{n+\frac{1}{2}}. \end{array}$$

Applying Cauchy inequality leads to

$$\begin{array}{cc} & \varepsilon \|r^{n+1}{\|}^{2}+\mu \|s^{n+1}{\|}^{2}\leq \varepsilon \|r^{n}{\|}^{2}+\mu \|s^{n}{\|}^{2}+\varepsilon \alpha_{t}{\left\|F^{n+\frac{1}{2}}\right\|}^{2}\\ & +\mu \alpha_{t}\|G^{n+\frac{1}{2}}{\|}^{2}+\frac{1}{2}\varepsilon \alpha_{t}(\|r^{n+1}{\|}^{2}+\|r^{n}{\|}^{2})+\frac{1}{2}\mu \alpha_{t}(\|s^{n+1}{\|}^{2}+\|s^{n}{\|}^{2}). \end{array}$$

So we derive by recursion that

$$\begin{array}{cc} & \varepsilon (1-\frac{\alpha_{t}}{2})\|r^{n+1}{\|}^{2}+\mu (1-\frac{\alpha_{t}}{2})\|s^{n+1}{\|}^{2}\leq \varepsilon (1+\frac{\alpha_{t}}{2})\|r^{0}{\|}^{2}+\mu (1+\frac{\alpha_{t}}{2}){\left\|s^{0}\right\|}^{2}\\ & +\varepsilon \alpha_{t}\Sigma_{k=1}^{n}\|F^{k+\frac{1}{2}}{\|}^{2}+\mu \alpha_{t}\Sigma_{k=1}^{n}\|G^{k+\frac{1}{2}}{\|}^{2}+\varepsilon \alpha_{t}\Sigma_{k=1}^{n}\|r^{k}{\|}^{2}+\mu \alpha_{t}\Sigma_{k=1}^{n}{\left\|s^{k}\right\|}^{2}. \end{array}$$

According to Gronwall inequality, we obtain the following error estimation:

$$\begin{array}{cc} & \varepsilon \|r^{n+1}{\|}^{2}+\mu {\left\|s^{n+1}\right\|}^{2}\leq \frac{2\alpha_{t}}{2-\alpha_{t}}\exp^{\frac{2n\alpha_{t}}{2-\alpha_{t}}}(\varepsilon \Sigma_{k=1}^{n}\|F^{k+\frac{1}{2}}{\|}^{2}+\mu \Sigma_{k=1}^{n}{\left\|G^{k+\frac{1}{2}}\right\|}^{2})\\ & +\frac{2+\alpha_{t}}{2-\alpha_{t}}\exp^{\frac{2n\alpha_{t}}{2-\alpha_{t}}}(\varepsilon \|r^{0}{\|}^{2}+\mu {\left\|s^{0}\right\|}^{2}). \end{array}$$ (41)

Under precise initial condition, (41) reduces to (38). By observing above inequality, we can find that, as the initial data are sufficiently accurate, LODEFMS scheme is convergent.

## Numerical examples

In this section, we apply our scheme LODEFMS scheme (22) to two Maxwell equations with periodic boundary condition and oscillatory solutions, to veirfy our theoretical analysis.

### One-dimensional example

We consider one-dimensional Maxwell equations (7) with the following oscillatory solution

$$\begin{array}{c} E_{z}=\sin(kx-\gamma t),\;H_{y}=-\beta E_{z},\;x\in \left[0,\;2\pi \right],\;t\in \left[0,\;10\right], \end{array}$$ (42)

where *ε* *γ* = *k* *β*, *μ* *β*^2^ = *ε*. Without loss of generality, here we take two sets of test data as follows:

1. *k* = *β* = *γ* = *ε* = *μ* = 1,
2. *k* = *γ* = 5, *β* = *ε* = *μ* = 1.

Select the step sizes as $\Delta t=0.01,\;\Delta x=\frac{\pi }{50}.$ Other step sizes have same numerical behavior.

**Fig 2:**
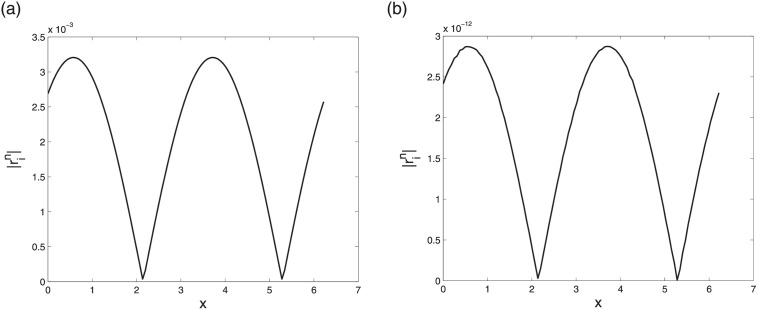
Numerical errors of central box scheme (left) and LODEFMS scheme (right) under first set of test data.

**Fig 3:**
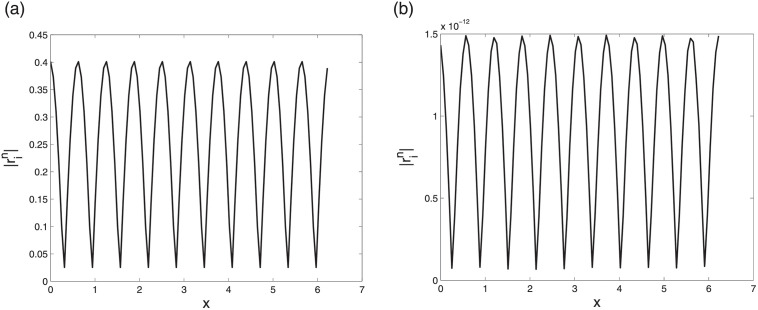
Numerical errors of central box scheme (left) and LODEFMS scheme (right) under second set of test data.

**Fig 4:**
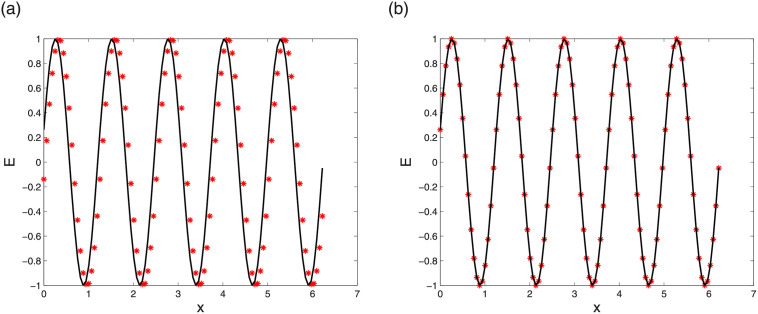
Waveform curves of exact solution (solid line), numerical solutions (star point) of central box scheme (left) and LODEFMS scheme (right) under second set of test data.

First we check the convergence by comparing the results of LODEFMS scheme and central box scheme to solve above one-dimension Maxwell equations with oscillatory solution (42). Figs 2 and 3 show error curves at *t* = 10 of central box scheme and LODEFMS scheme in two testing cases, respectively. We can see that, the errors of LODEFMS scheme are smaller than those of central box scheme. Waveform curves of exact oscillatory solution and numerical solutions of both schemes, at *t* = 10 under second set of test data, are depicted in Fig 4. It can be seen that, numerical solution of central box scheme is obviously different from the exact solution, while numerical solution of LODEFMS scheme agrees well with exact solution. LODEFMS scheme is more effective to simulate one-dimension Maxwell equations with oscillatory solutions.

**Fig 5:**
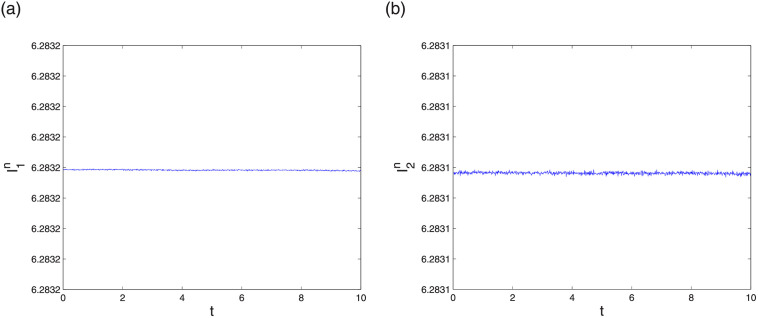
Discrete energy $I_{1}^{n}$ (left) and $I_{2}^{n}$ (right) in first testing case.

**Fig 6:**
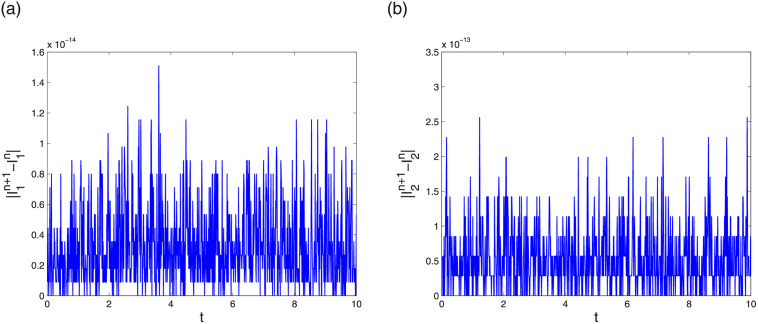
Errors of discrete energy $I_{1}^{n}$ (left) and $I_{2}^{n}$ (right) in second testing case.

Next analyse the conservation results of LODEFMS scheme. Fig 5 plots two discrete energy $I_{1}^{n}$ and $I_{2}^{n}$ in first testing case. The curves in the figure are similar to horizontal straight line. Error evolution curve at adjacent time of two discrete energy in second testing case is depicted in Fig 6. The error magnitude of $I_{1}^{n}$ and $I_{2}^{n}$ are approximately 10^−14^ and 10^−13^, respectively. This verifies that $I_{1}^{n}$ and $I_{2}^{n}$ are two conservation quantity of LODEFMS scheme.

Least square method is an effective tool to solve inverse problem of parameter estimation [35]. Next, we adopt least square method and LODEFMS scheme to fit the parameter *ε* in one-dimensional Maxwell equations. Measured data in simulation is theoretical solution with random disturbance *δ*. Suppose that random perturbation *δ* obeys a normal distribution with expectation 0 and variance *σ*. Now given simulation data at points $\left(x_{i},t_{k}\right),E_{z}^{i,k}=E_{z}\left(x_{i},t_{k}\right)+\delta ,H_{y}^{i,k}=H_{y}\left(x_{i},t_{k}\right)+\delta ,i=0,1,\cdots ,N_{x},k=0,1,\cdots ,N_{t},$ we choose parameter ε*, such that

$$\begin{array}{c} \arg\min_{\varepsilon }\Sigma_{i=0}^{m_{1}}\Sigma_{k=0}^{m_{2}}\left\{{\left[E_{z}^{i,k}-E_{zi}^{k}\left(\varepsilon \right)\right]}^{2}+{\left[H_{y}^{i,k}-H_{yi}^{k}\left(\varepsilon \right)\right]}^{2}\right\}=\varepsilon^{*}, \end{array}$$ (43)

where $E_{zi}^{k},H_{yi}^{k}$ are our numerical solutions. Assume that the real parameter in experiment is *ε* = 1.

**Fig 7:**
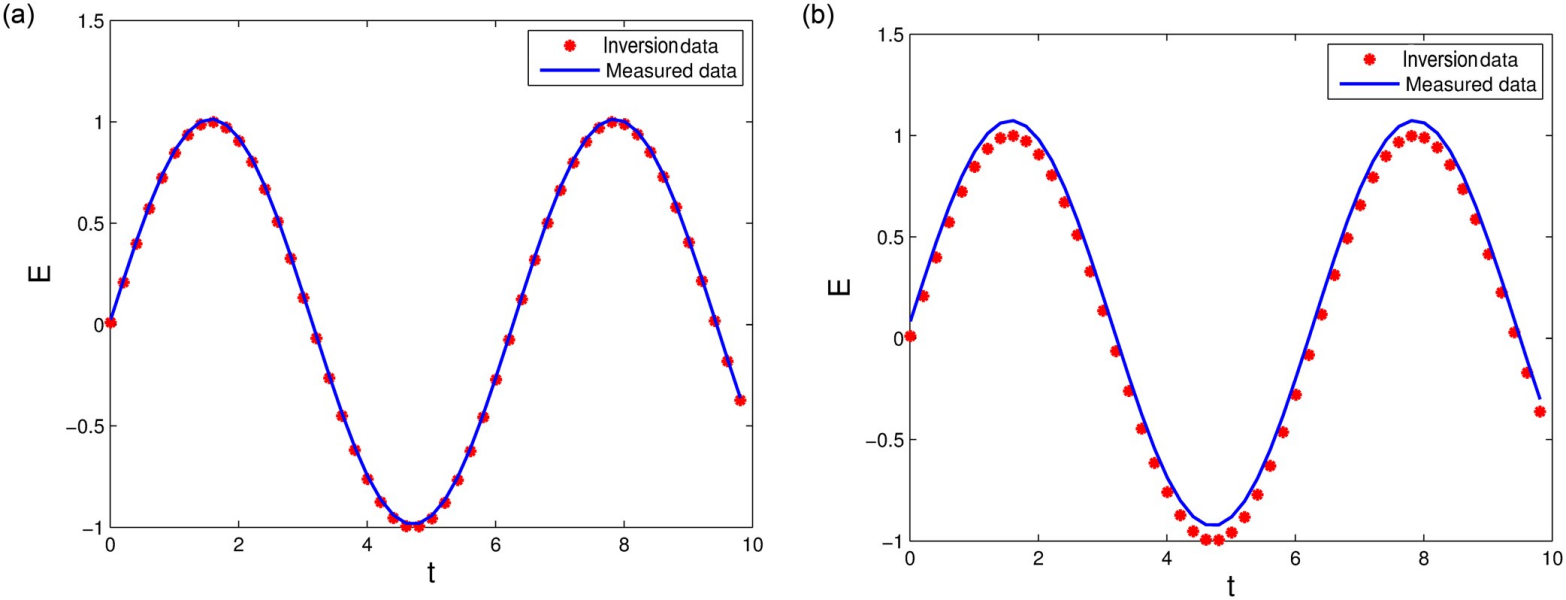
Measured data and inversion data under disturbance *σ* = 0.01 (left) and *σ* = 0.05 (right).

**Fig 8:**
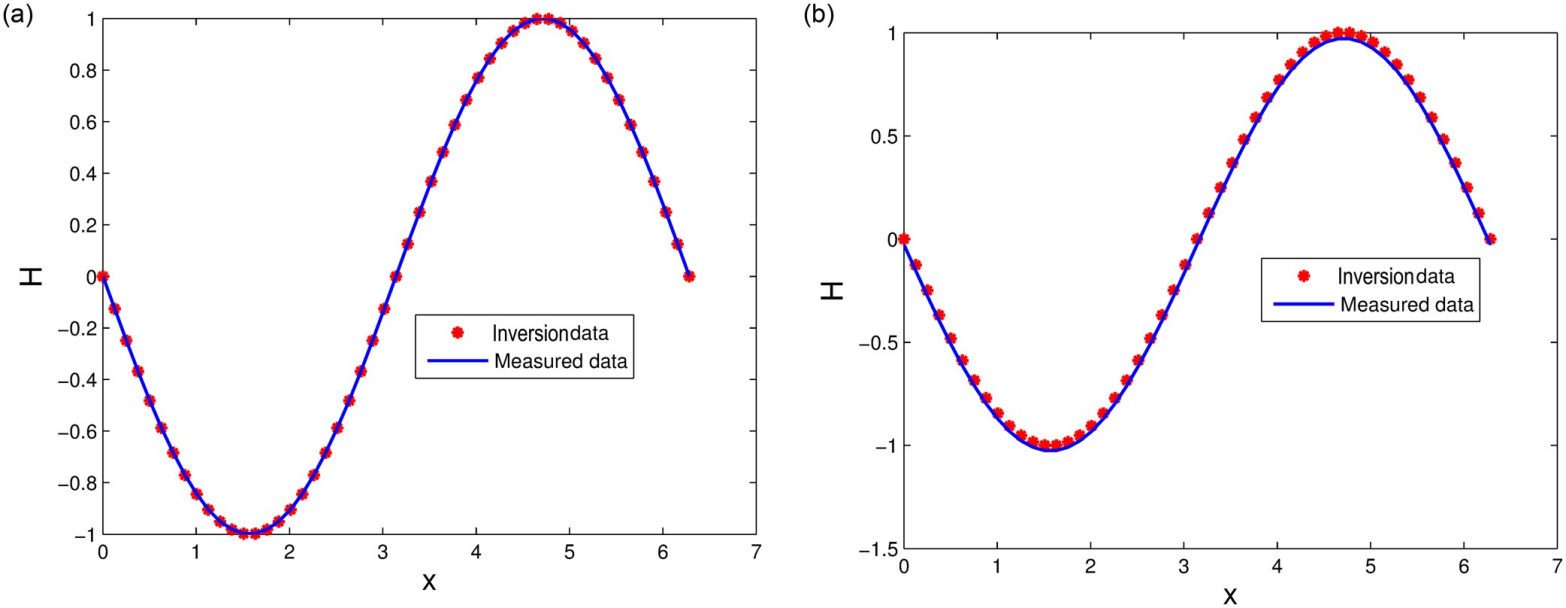
Measured data and inversion data under disturbance *σ* = 0.004 (left) and *σ* = 0.04 (right).

Table 1 lists the inverting parameter ε* under different distribution *σ*. Figs 7 and 8 show measured data and inversion data with *x* = *π*, *t* ∈ [0, 10] and *x* ∈ [0, 2*π*], *t* = 0.5, respectively. The results show that the inversion data is consistent with measured data. The inverting parameters estimated by least square method and LODEFMS scheme are reasonable under small disturbance error.

**Table 1:** Inverting parameter ε* under different distribution *σ*.

*σ*	0.001	0.005	0.01	0.05
ε*	0.9999	1.0003	1.0011	0.9953

### Three-dimensional example

For sake of simplicity, we apply LODEFMS scheme (22) to solve three-dimensional Maxwell equations (1) with oscillatory solution as follows:

$$\begin{array}{c} E_{x}=\cos[k(x+y+z)-\gamma t],\;E_{y}=-2E_{x},\;H_{x}=\beta E_{x},\;E_{z}=E_{x},\;H_{y}=0,\;H_{z}=-H_{x}, \end{array}$$ (44)

where *ε* *γ* = *k* *β*, *μ* *β*^2^ = 3*ε*. Generally, we adopt the following two sets of test data:

1.
$\varepsilon =\mu =1,\;k=2\pi ,\;\beta =\sqrt{3},\;x,y,z\in \left[0,\;1\right],$

2.
$\varepsilon =\mu =1,\;k=8,\;\beta =\sqrt{3},\;x,y,z\in \left[0,\;\pi /4\right].$

**Fig 9:**
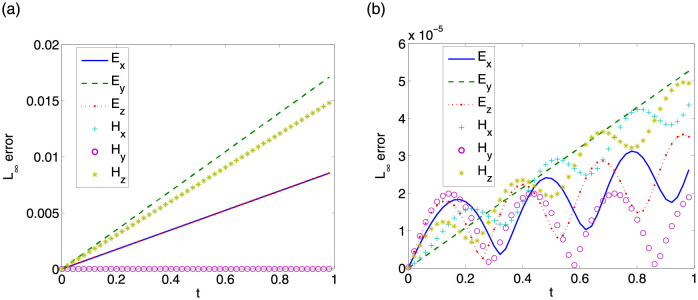
*L*_∞_ errors of numerical solutions to LOD central box scheme (left) and LODEFMS scheme (right) in first testing case.

**Fig 10:**
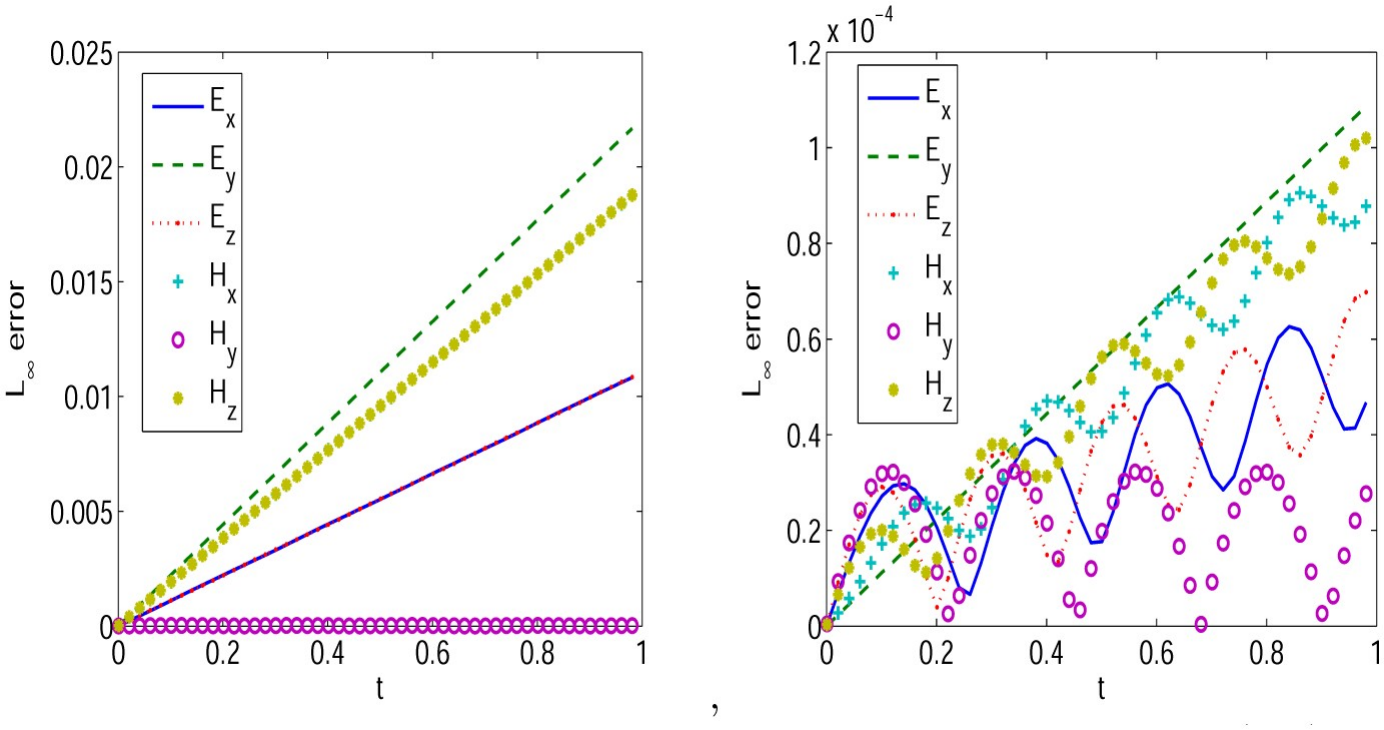
*L*_∞_ errors of numerical solutions to LOD central box scheme (left) and LODEFMS scheme (right) in second testing case.

**Fig 11:**
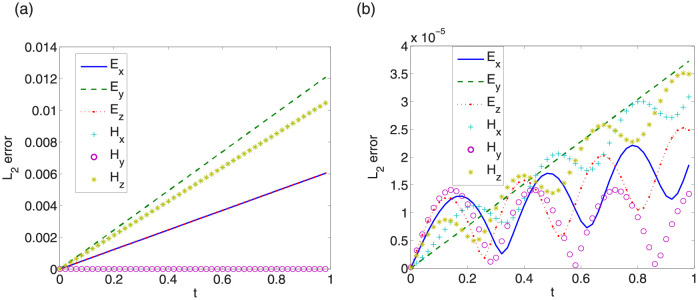
*L*_2_ errors of numerical solutions to LOD central box scheme (left) and LODEFMS scheme (right) in first testing case.

**Fig 12:**
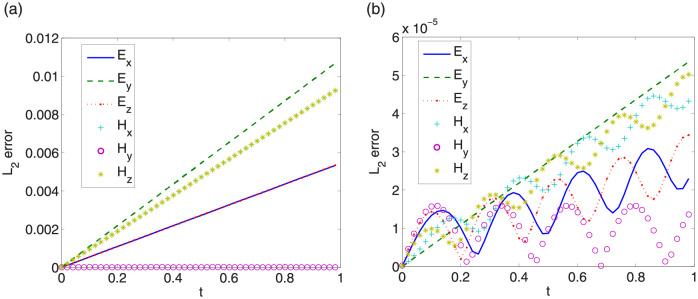
*L*_2_ errors of numerical solutions to LOD central box scheme (left) and LODEFMS scheme (right) in second testing case.

First, we give convergence results with step sizes $\Delta t=0.0001,\;\Delta x=\Delta y=\Delta z=\frac{\pi }{32k}.$ Same convergence behavior is obtained for other step sizes. *L*_∞_ errors between exact solutions and numerical solutions of LODEFMS scheme and LOD central box scheme are depicted in Figs 9 and 10 in above two cases, respectively. Figs 11 and 12 show *L*_2_ errors of numerical solutions. From the figures, we observe that the errors of LODEFMS scheme are smaller than those of LOD central box scheme.

**Fig 13:**
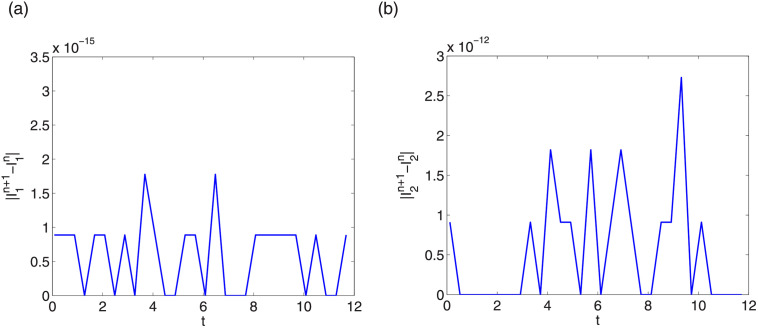
Errors of discrete energy $I_{1}^{n}$ (left) and $I_{2}^{n}$ (right) in first testing case.

**Fig 14:**
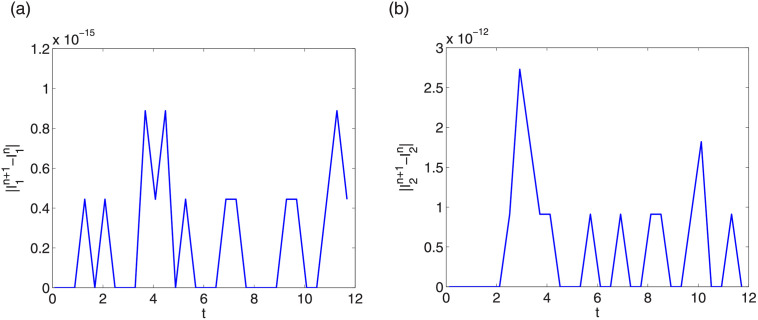
Errors of discrete energy $I_{1}^{n}$ (left) and $I_{2}^{n}$ (right) in second testing case.

Next we check energy conservation property of LODEFMS scheme. Generally, we take step sizes as $\Delta t=0.04,\;\Delta x=\Delta y=\Delta z=\frac{\pi }{4k}.$ Figs 13 and 14 show errors of discrete energy $I_{1}^{n}$ and $I_{2}^{n}$ in two testing cases, respectively. We find that, the error magnitudes of $I_{1}^{n}$ and $I_{2}^{n}$ are about 10^−15^ and 10^−12^, respectively. Same conservation results can be obtained by other step sizes. This verifies that LODEFMS scheme preserves two discrete energy conservation laws.

**Fig 15:**
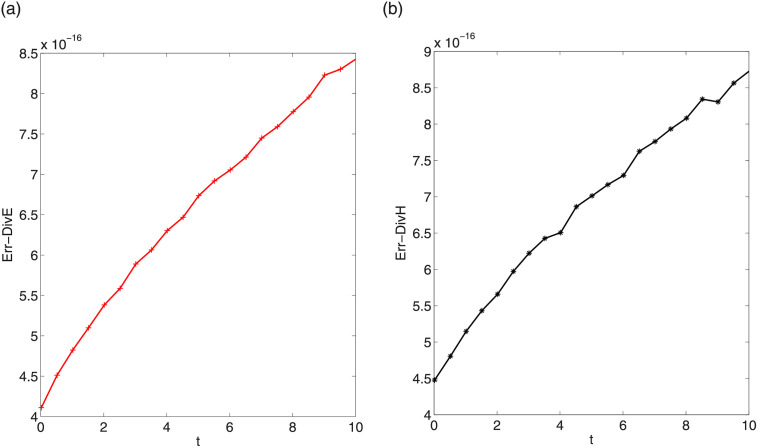
Divergence errors Err-DivE (left) and Err-DivH (right) in first testing case.

**Fig 16:**
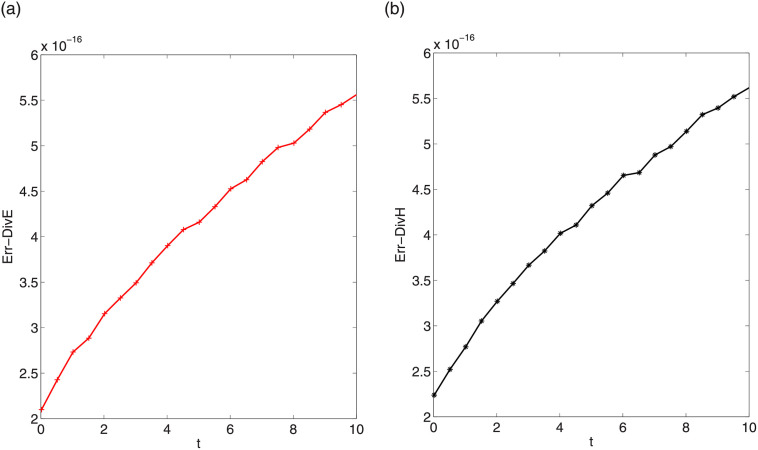
Divergence errors Err-DivE (left) and Err-DivH (right) in second testing case.

At last, to test the conservation of discrete divergence, we define the errors of two discrete divergences by

$$\text{Err-DivE}\left(n\right)=\Delta x\Delta y\Delta z\sum_{i,j,k}\left|{\bar{\nabla }}_{i,j,k}\cdot E^{n+1}-{\bar{\nabla }}_{i,j,k}\cdot E^{n}\right|,$$

$$\text{Err-DivH}\left(n\right)=\Delta x\Delta y\Delta z\sum_{i,j,k}\left|{\bar{\nabla }}_{i,j,k}\cdot H^{n+1}-{\bar{\nabla }}_{i,j,k}\cdot H^{n}\right|,$$

respectively. Figs 15 and 16 display the divergence errors with step sizes $\Delta t=0.01,\;\Delta x=\Delta y=\Delta z=\frac{\pi }{32k}.$ We observe that the scale of the errors is about 10^−16^. In other data cases, we can obtain the same conservation behavior of discrete divergences.

## Conclusion

Conservative Maxwell equations with periodic oscillatory solutions are investigated numerically. First we have constructed a conservative multisymplectic scheme, preserving two discrete energy invariants and two discrete divergences, by applying exponentially fitted trapezoidal scheme to solve Maxwell equations with periodic solutions. Then LOD multisymplectic scheme is presented in order to reduce the cost of calculation. Unconditional stability, dispersion and convergence analysis for LODEFMS scheme are established additionally. We have carried out two examples in simulating Maxwell equations with periodic oscillatory solutions to illustrate the effectiveness and conservation property of LODEFMS scheme. In addition, taking one-dimensional Maxwell equation as an example, we find that least square method and LODEFMS scheme can be combined to fit the electric permittivity under small random perturbation data.
